# The safety of health care for ethnic minority patients: a systematic review

**DOI:** 10.1186/s12939-020-01223-2

**Published:** 2020-07-08

**Authors:** Ashfaq Chauhan, Merrilyn Walton, Elizabeth Manias, Ramesh Lahiru Walpola, Holly Seale, Monika Latanik, Desiree Leone, Stephen Mears, Reema Harrison

**Affiliations:** 1 School of Public Health and Community Medicine, University of New South Wales, Sydney, 2052 NSW Australia; 2 School of Public Health, University of Sydney, Sydney, 2006 NSW Australia; 3 School of Nursing and Midwifery, Centre for Quality and Patient Safety Research, Institute for Health Transformation, Deakin University, Melbourne, 3025 VIC Australia; 4 Multicultural Health, Western Sydney Local Health District, Westmead, 2145 NSW Australia; 5 Hunter New England Health Libraries, Hunter New England Local Health District, Tamworth, 2310 NSW Australia

**Keywords:** Patient safety, Patient safety events, Inequity, Ethnic minority, Healthcare disparities, Patient engagement

## Abstract

**Introduction:**

Evidence to date indicates that patients from ethnic minority backgrounds may experience disparity in the quality and safety of health care they receive due to a range of socio-cultural factors. Although heightened risk of patient safety events is of key concern, there is a dearth of evidence regarding the nature and rate of patient safety events occurring amongst ethnic minority consumers, which is critical for the development of relevant intervention approaches to enhance the safety of their care.

**Objectives:**

To establish how ethnic minority populations are conceptualised in the international literature, and the implications of this in shaping of our findings; the evidence of patient safety events arising among ethnic minority healthcare consumers internationally; and the individual, service and system factors that contribute to unsafe care.

**Method:**

A systematic review of five databases (MEDLINE, PUBMED, PsycINFO, EMBASE and CINAHL) were undertaken using subject headings (MeSH) and keywords to identify studies relevant to our objectives. Inclusion criteria were applied independently by two researchers. A narrative synthesis was undertaken due to heterogeneity of the study designs of included studies followed by a study appraisal process.

**Results:**

Forty-five studies were included in this review. Findings indicate that: (1) those from ethnic minority backgrounds were conceptualised variably; (2) people from ethnic minority backgrounds had higher rates of hospital acquired infections, complications, adverse drug events and dosing errors when compared to the wider population; and (3) factors including language proficiency, beliefs about illness and treatment, formal and informal interpreter use, consumer engagement, and interactions with health professionals contributed to increased risk of safety events amongst these populations.

**Conclusion:**

Ethnic minority consumers may experience inequity in the safety of care and be at higher risk of patient safety events. Health services and systems must consider the individual, inter- and intra-ethnic variations in the nature of safety events to understand the where and how to invest resource to enhance equity in the safety of care.

**Review registration:**

This systematic review is registered with Research Registry: reviewregistry761.

## Introduction

A multitude of factors contribute to health inequity amongst ethnic minority populations including limited social support, lower health literacy, lower socio-economic status, greater incidence of ill health and a sense of disempowerment [1–4]. Access to care and language barriers have been the predominant focus of research, with evidence of failure to provide qualified interpreting services to people with limited English proficiency (LEP) as a key contributor to poor care outcomes [5–12].

Whilst health inequities amongst ethnic minorities internationally are well-established, patients’ ethnicity, language and culture are increasingly recognised as significant predictors of the quality of health care delivery in addition to health outcomes [13–16]. An emerging body of research indicates that patients from minority groups are at higher risk of patient safety events,which are events that could have or did result in harm to the patient, compared to the mainstream population [17–19].

Internationally, there has been significant investment in enhancing patient safety mechanisms across health systems [20] through funded international and national bodies responsible for patient safety in health, including dedicated units within the World Health Organisation (WHO) and organisations such as the Australian Commission on Safety and Quality in Health Care (ACSQHC) in Australia, the National Safety Investigation Branch in the UK and the Agency for Healthcare Research and Quality (AHRQ) in the US. Widespread implementation of clinical governance frameworks by these bodies and human capital and system-level resourcing to improve safety in care such as incident reporting and analysis among others has been notable over the past 30 years [20–23].

Despite international efforts to enhance patient safety and care quality, there has been limited focus on improving safety for ethnic minority populations, which remains an under-researched area [13, 24]. Patients from ethnic minorities continue to report feeling unsafe when receiving health care, with experiences of discrimination, not having appropriate interpreting services and having inadequate knowledge of healthcare settings [25]. Current policy guidelines in Australia and many countries internationally require use of trained interpreters, yet research to date highlights patients reliance on informal translating mediums to support their healthcare interactions [25]. Lack of sufficient systems support comprising inadequate policies and resource for mandatory use of trained interpreters may lead to poor quality interactions between patients, carers and professionals and exacerbate the risk of safety events [17].

In the context of established disparities in the rate of safety events experienced by ethnic minority and majority populations, the nature and rate of patient safety events and the underpinning factors associated with safe care involving ethnic minorities are not well understood [17]. Systematic reviews of patient safety events for ethnic minorities have been limited to only one type of patient safety event, e.g. medication errors or country [26]. To address the knowledge gap, we conducted a systematic review with the following objectives:1) how ethnic minority populations are conceptualised across the international literature and the implications for this conceptualisation in shaping our findings; 2) to establish the evidence for patient safety events involving ethnic minority healthcare consumers internationally; and 3) the individual, service and system factors that contribute to safety among ethnic minority healthcare consumers.

## Methods

The Preferred Reporting Items for Systematic Review and Meta-Analyses (PRISMA) statement was used to guide the reporting of this systematic review [27].

### Inclusion criteria

We included available publications in English that reported original, primary empirical, conceptual or theoretical work published from January 2000 to October 2019. Conceptual, theoretical, quantitative or qualitative studies of any research design were eligible including systematic reviews. Studies had to include a sub- or full sample of ethnic minority patients/consumers or data related to ethnic minority patients/consumers. Ethnic minority patients/consumers were defined broadly to include any group who did not speak one of the national languages of the study country, were born in another country, or those who belonged to an ethnic minority group. Outcomes relating to any patient safety events were included except for the specific exclusion applied below. We defined patient safety event as an event that could have or did result in unnecessary harm to the patient.

### Exclusion criteria

Publications were excluded if they were not from countries within the Organisation for Economic Co-operation and Development (OECD). Case reports, letters, editorials, and comments were excluded. Studies discussing the following were also excluded: 1) disparity in clinical outcomes or health outcomes in ethnic minorities; 2) diagnostic disparities in ethnic minorities in mental health settings; 3) disparities in access to health care, health prevention and health promotion activities; 4) disparity in quality of healthcare delivery; and 5) safety issues or incidents relating to multicultural healthcare staff. Though these studies are important in understanding the health disparities between ethnic minorities and the mainstream population, they present their own line of inquiry and were considered too broad for this systematic review.

### Study identification

An initial range of text words, synonyms and subject headings for the two concepts of this study, patient safety events and ethnic minority, were compiled using several documents as a guide [18, 28, 29]. A lack of consistent terminology used across studies meant that a broad range MeSH (Medical Subject Headings) terms and text words were needed to cover each concept. For example, the concept of patient safety events can be encompassed by subject headings and text words such as adverse events, medication errors, diagnostic errors, as well as patient safety, among others. The concept of an ethnic minority is encompassed by an even wider range of subject headings and text words including cultural diversity, ethnic groups, minority groups and Non-English Speaking. We searched five databases (MEDLINE, PUBMED, PsycINFO, EMBASE and CINAHL). We also hand searched the reference list of the included studies to ensure that we included all relevant studies.

### Study selection and data extraction

The database search was uploaded to Covidence systematic review software (Veritas Health Innovation, Melbourne, Australia) with articles available online for initial title and abstract review. Based on the information available in the title and abstract, one reviewer (AC) completed the initial title and abstract review. The inclusion criteria were then independently applied for full text review by two reviewers (AC; RH). Disagreements were resolved by consultation. The following data were extracted from included studies: author, publication year, location & setting, research methodology & sample, population studied, objectives and key findings.

### Assessment of study quality

Due to heterogeneity of the study types included, we used a revised version of the Quality Assessment Tool for Studies with Diverse Designs (QATSDD) with 13 items [30]. The revised version was checked for reliability with two reviewers independently scoring the included studies. The Kappa test was used for inter-rater reliability [31]. The score of 0.65 was obtained and is considered substantial for reliability [31, 32].

### Narrative data synthesis

We used a narrative synthesis to analyse our findings due to the heterogeneity of the study types, as a pure quantitative or qualitative approach for synthesis was not suitable. In addition, the outcomes discussed in each study were not directly comparable. The data extraction provided us with key findings from each study. A textual approach was used to summarise and synthesise the findings from the included studies against the stated objectives.

## Results

**Fig. 1:**
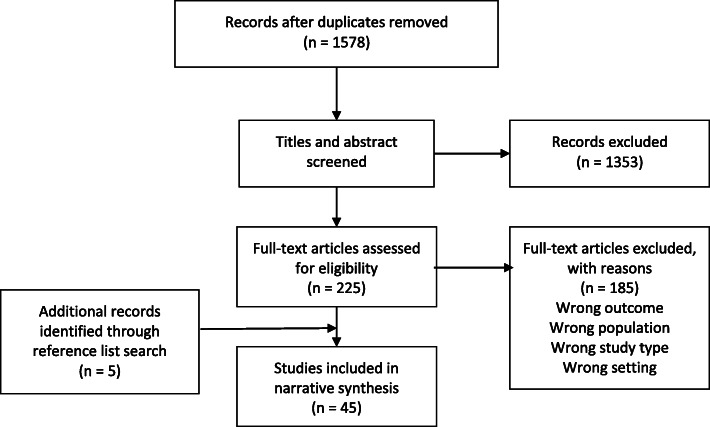
PRISMA Flow Diagram

After removing duplicates, 1578 articles were identified. Initial title and abstract screening led to identification of 225 studies. Based on the inclusion and exclusion criteria, two reviewers then reviewed the full text which resulted in 40 studies for inclusion. The reference list searching of the included articles led of inclusion of additional 5 studies. See PRISMA diagram for full search strategy (Fig. 1).

### Study characteristics

The included studies represented various countries, health settings and a range of study methods. The studies originated from the United States (US) (25), The Netherlands (5), The United Kingdom (UK) (2), Australia (5), Canada (1), Denmark (1), Switzerland (1), Sweden (1), and Israel (1). Three studies did not specify a geographic setting. A large number of studies were based in hospital settings (26) with others based in the community such as a pharmacy (7), and primary health setting (5). Two studies did not specify a setting. A majority of the included studies used large administrative datasets or retrospective chart review (14) followed by qualitative studies conducting explorative analysis of patient safety events (8) and systematic reviews (8). Summary of the study findings are presented in Table 1.

**Table 1:** Summary of study findings

Authors	Year	Setting/ Country	Method/ Sample	Ethnic minority conceptualisation	Aims/Objectives	Relevant Findings
Alhomoud et al [26]	2013	UK Community setting	Systematic Review *n* = 15 articles	Ethnic minorities. Defined as groups who share minority status in their country of residence due to ethnicity, place of birth, language, religion, citizenship and other cultural differences.	a) Establish types and causes of medicine related problems (MRPs) b) Identify recommendations in support of effective use of these medications.	• Differing cultural perceptions and beliefs about health, illness, prescribed treatment and medical care impact on the use of medicine. • Lack of awareness of the extent of patients’ decision-making regarding the use of their medicines and/or poor appreciation of their experience of MRPs, may cause MRPs • Recommendations identified involved education, use of interpreters, bilingual workers to bridge the cultural divide and to encourage participation of minority communities in decision-making.
Alhomoud et al [33]	2015	UK Community setting (pharmacy)	Face to face semi-structured interviews *n* = 80	South Asian and Middle Eastern patients	a) To describe medicine related problems b) Identify contributing factors	• Religious practices and beliefs, use of non-prescription medicines, extent of family support, and travelling abroad--to patient’s homeland or to take religious journeys were identified as factors specific to SA and ME patients. • Illiteracy, language and communication barriers, lack of translated resources, perceptions of healthcare providers, and difficulty consulting a doctor of the same gender may also contribute to MRPs.
Ajdukovic et al [34]	2007	Australia Hospital setting (ED)	Interviews with patients. *n* = 100 patients (24 with language barrier)	Patient subgroups; ‘general’ patients, patients with a ‘language barrier’ and patients from a residential aged care facility.	a) To identify medication-related ED presentations and describe the incidence in these demographic groups.	• The number of correctly recorded medications was lowest in the ‘language barrier’ group (13.8%) compared with 18 and 19.6% of medications for ‘general’ patients and patients from residential aged care facilities respectively.
Baehr et al [35]	2015	US All settings	Systematic Review *n* = 40 articles.	Low English Proficiency (LEP)	a) To synthesize existing literature in order to understand the state of evidence for racial and ethnic disparities associated with ADEs in the USA b) To identify gaps in existing knowledge in order to target future research.	• Asian race was associated with an approximately fourfold risk of anticoagulant related ADEs such as bleeding, haemorrhage. • African Americans were the most commonly identified group at risk for ADEs caused by diabetes agents such as hypoglycaemia. • Caucasians were most frequently identified as at risk for opioid related ADEs such as overdose. • Opioid related side effects were noted in 58% of African Americans as compared to Caucasians.
Bakullari et al [15]	2015	US Hospital Setting	Retrospective chart review *n* = 79,019	Six racial/ethnic groups.	a) To determine whether racial/ethnic disparities exist in the rate of occurrence of Healthcare Acquired Infections captured in the Medicare Patient Safety Monitoring System (MPSMS).	• The occurrence rate for HAIs was 1.1% for Caucasians, 1.3% for African Americans, 1.5% for Hispanic patients, 1.8% for Asian patients, 1.7% for Native Hawaiian/Pacific Islander patients, and 0.70% for other patients. • Compared with Caucasians patients, the odds ratios of occurrence of HAIs were 1.1 (95% confidence interval [CI], 0.99–1.23) for African Americans, 1.3 (95% CI, 1.15–1.53) for Hispanics, 1.4 (95% CI, 1.07–1.75) for Asians.
Blennerhasset et al [36]	2011	Australia Community Setting (Pharmacy)	Multistage qualitative study Interviews with patients (*n* = 18) and focus groups (*n* = 9) with staff.	Greek [5], Russian [5], mandarin or Cantonese [4] and English [4] speaking.	a) Examine medicine management in older people from Non-English-speaking background.	• Patients lack knowledge about medications and medication changes. • Interpreter services not routinely used. • Lack of systematic, standardized processes for identifying people at risk of medicine mismanagement or for the implementation of actions to minimise risks.
Bloo et al [37]	2014	Country not applicable Hospital setting	Systematic review *n* = 26	Ethnic minorities	a) Review the literature on safety differences between patients from minority ethnic groups and those from the ethnic majority undergoing surgery.	• Minority ethnic groups had statistically significant higher complication rates compared with the ethnic majority. • Higher incidence of infection and graft rejection among ethnic minorities • Higher incidence of pain and re-operation among ethnic minorities.
Cantarero-Arevalo et al [38]	2014	Denmark Community setting	Before and after study. Mixed Methods. Participants: Pre *n* = 30 Post *n* = 23	Arabic speaking from 10 different countries.	a) To explore the perceptions, barriers and needs of Arabic-speaking ethnic minorities regarding medicine use b) To use an education program to enhance the knowledge and competencies of the ethnic minorities about the appropriate use of medicines	• Misconceptions relating to medication use, mistrust with Danish doctors and low compliance to doctor’s recommendations were identified as barriers to correct medicine use. • A culturally competent education program may potentially reduce medicine-related problems.
Coffey et al [39]	2005	US Hospital setting	Quantitative Study Sixteen Sate sample.	Three racial/ethnic minorities group (Black, Hispanics & Asian/Pacific Islanders)	a) To determine whether racial and ethnic differences between in patient safety events disappear when income is considered.	• Each ethnic minority group has a higher rate of nosocomial infections, post-operative sepsis and 2 post-operative complications. • African Americans have 1.25 to 1.5 times the rate of infection due to medical care, postoperative sepsis, decubitus ulcers, postoperative respiratory failure and PE/DVT as Caucasians. • Hispanics have 1.25 to 1.50 times the rate of 2 post-operative complications as Caucasians. • Asian/Pacific Islanders have 1.25 to 1.50 times the rate of 4 post-operative complications.
Cohen et al [40]	2005	US Hospital setting: Paediatric.	Case control study Cases, *n* = 97 Ctrl, *n* = 475	Language barrier defined by self- or provider-reported need for an interpreter.	a) Determine whether hospitalized paediatric patients whose families have language barriers are more likely to incur serious medical errors than patients whose families do not have language barriers	• No increased risk for serious medical events in patients and families who requested an interpreter compared with patients and families who did not request an interpreter (odds ratio: 1.36; 95% confidence interval: 0.73–2.55). • Spanish speaking patients who requested an interpreter had two-fold increased risk of adverse events compared with patients who did not request an interpreter. • All other language groups composed of < 1% patient population and had no detectable increased risk of adverse events.
Divi et al [41]	2007	US Hospital setting.	Quantitative analysis of AEs incident reports *n* = 1083	Low English proficiently – LEP	a) To examine differences in the characteristics of adverse events between English speaking patients and patients with limited English proficiency in US hospitals.	• Overall, 29.5% of reported adverse events in English speaking patients and 49.1% of reported adverse events in LEP patients caused some physical harm to the patient • LEP patients experienced a statistically significantly greater proportion of adverse events that were attributable to communication failure (52.4%) than did English speaking patients (35.9%). • LEP patients experienced a statistically significantly greater proportion of events attributable to questionable advice/interpretation than English speaking patients (11.2 vs. 3.5%). • Overall, system factors were found to play a statistically significantly greater role in the occurrence of adverse events for LEP patients than for English speaking patients especially, they were more attributable to organisational factors. • Adverse events associated with practitioner factors occurred more often to LEP patients than to English speaking patients.
Flores et al [42]	2003	US Hospital setting: Paediatrics	Analyses of audiotaped interpreter encounters *n* = 13	Spanish speaking	a) To determine the frequency, categories and potential clinical consequences of errors in medical interpretation.	• Sixty three percent of all errors had a potential clinical consequence. • Errors committed by ad hoc interpreters were more likely to result in a significant clinical consequence as compared to hospital interpreters. • Errors of clinical consequence included: 1) omitting questions about drug allergies; 2) omitting instructions on the dose, frequency, and duration of antibiotics and rehydration fluids; 3) adding that hydrocortisone cream must be applied to the entire body, instead of only to facial rash; 4) instructing a mother not to answer personal questions; 5) omitting that a child was already swabbed for a stool culture; and 6) instructing a mother to put amoxicillin in both ears for treatment of otitis media.
Flores et al [43]	2012	US Hospital setting: Paediatrics	Analyses of audiotaped encounters (n = 57), 20 with professional interpreters, 27 with ad hoc interpreters and 10 with no interpreters.	Spanish speaking limited English proficiency patients, caregivers, families and their interpreters	a) To compare interpreter errors and their potential consequences in encounters with professional vs ad hoc vs no interpreters.	• The proportion of errors of potential consequence was significantly lower for professional (12%) versus ad hoc (22%) versus no interpreters (20%). • Among professional interpreters, previous hours of interpreter training, but not years of experience, were significantly associated with error numbers, types, and potential consequences. • Interpreters with greater than or equal to 100 h of training committed significantly lower proportions of errors of potential consequence overall (2% versus 12%)
Fejzic et al [44]	2004	Australia Community Setting	Mixed method *n* = 25 NESB patients	NESB – residents of former Yugoslavia now residing in Australia.	a) Medication management reviews (MMR) in people from a NESB in their native language in order to identify medication-related problems	• Psychological and sociological factors were identified as having significant impacts on medication management. • Not understanding how to take medications, unknown side effects, no recognition of professional help, taking paracetamol wrong were identified as some of the main medication related problems.
Goenka [45]	2016	US Not specified	Literature Review Sample size not available.	Low English Proficiency (LEP) patients	a) To summarize the legal basis for providing language access in the healthcare setting, discuss the impact of interpretation services on clinical care, and explore the effects of language barriers on health outcomes	• There is often an overestimation of patients’ English proficiency leading to inadequate language assistance. • The lack of interpretation, or use of informal, untrained interpreters, has significant effects on patient safety, quality of care, and patient satisfaction. • The inconsistencies in communication appear to have a direct impact on the incidence of adverse events. • Children with LEP parents are twice as likely to experience a preventable adverse drug event.
Hadziabdic et al [46]	2011	Sweden Community setting: primary health care	Qualitative analysis of incidents (*n* = 60 incidents)	Person of foreign background.	a) To explore what problems are reported by professionals in primary healthcare concerning the use of interpreters and what the problems lead to.	• Incident reports analysis highlighted problems faced by health care professionals due to lack of available interpreters and also lack of interpreters’ understanding of Swedish. • The main problems documented were related to language, such as lack of the interpreters with proficiency in a particular language, and to organisational routines, with difficulties in the availability of interpreters and access to the interpreter agency. • Consultations were limited possibilities to communicate and thus consultation was carried out without a professional interpreter, using family members instead.
Harris et al [47]	2017	US Outpatient clinical setting – Paediatrics.	Cross-sectional analysis of data from multisite randomized controlled experiment *n* = 1126	Self-identified as Hispanics	a) To examine associations between health literacy, LEP, and liquid medication dosing errors in Hispanic parents.	• Liquid medication dosing errors by Hispanic parents are common, with over 80% of parents making at least one error, and that errors were more common among those with limited health literacy and LEP. • Efforts to revise existing standards or to redesign paediatric medication labels and dosing tools should be specifically tailored to meet the needs of limited literacy and LEP individuals.
Hernandez-Suarez et al [48]	2019	US Hospital Setting	National Inpatient Sample (NIS) database files *n* = 36,270	Caucasians, African Americans and Hispanics.	a) To identify racial/ethnic disparities in utilization rates, in-hospital outcomes and health care resource use among Non-Hispanic Whites (NHW), African Americans (AA) and Hispanics undergoing TAVR	• Hispanic patients had higher in-hospital complications. • Hispanic was associated with higher incidence of acute myocardial infarction (aOR = 2.02; 95%CI, 1.06–3.85;*P* = .03), stroke/transient ischemic attack (aOR = 1.81; 95% CI, 1.04–3.14;*P* = .04), acute kidney injury (aOR = 1.65; 95% CI, 1.23–2.21;Pb.01).
Inagaki et al [49]	2017	US Hospital setting	Retrospective record review (Patients: Non-English Speaking - 51, English speaking – 210)	Non-English Speaking	a) To evaluate the effect of language discordance on post-operative outcomes among vascular surgery patients.	• Adjusted analysis showed that language discordance did not affect the odds of adverse outcomes of wound infections or adverse graft events.
Karliner et al [50]	2012	US Hospital Setting	Participant survey followed by a phone interview *n* = 308 participants	LEP – Low English Proficiency (English, Spanish and Chinese)	a) Was language barrier is associated with lower rates of understanding of discharge instructions, including diagnosis, type of follow-up appointments, and medication category and purpose after discharge from the acute care hospital b) Was language concordance and interpretation at discharge associated with understanding of discharge instructions.	• Most LEP patients’ understanding of medications and of the type of follow-up appointment was low. • Language concordant discharge instructions had lower odds of understanding for outcomes related to appointment type and combined medication category and purpose. • LEP participants reporting a family/friend interpreter at discharge had lower odds of understanding their medications. • LEP participants reporting no interpretation at discharge had similar outcomes to the English-speaking group. • Number of medications was associated with lower rates of medication understanding regardless of other factors and each additional medication was associated with a 10–15% reduction in rate of any kind of medication understanding.
Koster et al [51]	2014	The Netherlands Community setting	Cross-sectional study (quantitative survey) of 691 non-Dutch speaking migrants.	First generation migrants. *(Antilles, Persians, Surinamese and Turks)	a) Assess interpretation of drug label instructions in different migrant populations living in the Netherlands.	• Many standardly used drug label instructions are unclear, and misinterpretation of these instructions occurs both in highly educated natives and immigrants. • Turkish migrants most often experienced problems withcomprehension of the tested instructions. • Information presented on these labels is very limited, and these labels are only effective if patients are able to understand instructions, interpreted the information correctly, and can follow the advice provided on them. • Lower understanding of drug labels for Surinamese and Antillean migrants compared to the reference group was not observed as Dutch is also an official language in these countries.
Lee et al [52]	2015	US Community Setting	A pre- postsurvey study. (*n* = 68)	Korean adult migrants	a) Investigate the knowledge and understanding of older adult Korean immigrants concerning prescription and over-the-counter medication directions.	• Even when information is communicated in native language, there is a lack of understanding of medication directio
Lion et al [53]	2013	US Hospital setting: Paediatrics	Retrospective chart review of 33,885 admissions	Self-reported primary language spoken was used to identify participants because no data on English proficiency were available for the study period.	a) To evaluate the risk for serious/sentinel adverse events among hospitalized children according to race, ethnicity, and language and to evaluate factors affecting length of stay associated with serious/sentinel adverse events.	• Hospitalized children from Spanish speaking families had significantly longer hospital stays in association with an adverse event and may have increased odds of a serious or sentinel event. • Results according to ethnicity were similar but somewhat attenuated, suggesting that language difference, rather than Hispanic/Latino ethnicity, was the operative factor.
Lopez et al [54]	2015	US Hospital setting	Retrospective cohort study *n* = 4,224,564 (13%) were LEP.	Self-reported LEP status	a) Are hospitalized LEP patients receiving interpreter services during hospital clinical encounters?	• Results indicate that in a well-resourced academic hospital, use of interpreters by clinical staff remains highly variable, with 66% of LEP patients having no interpreter use during the inpatient clinical encounter.
Masland et al [55]	2011	US Community setting	Cross-sectional survey *n* = 48,968 surveys	Mexicans, Central Americans, Chinese, Koreans, and Vietnamese.	a) Examine the effect of language and cultural factors on prescription label understanding by ethnic groups.	• In multivariate analysis, limited English increased odds of difficulty in understanding prescriptions by three times for Mexicans, Central Americans, and Koreans, and four times for Chinese; it was insignificant for Vietnamese. • In controlled analysis, Chinese and Korean ethnicity increased odds of difficulty of understanding compared to Mexican or Central American ethnicity; Vietnamese ethnicity reduced odds of difficulty compared to others. • Having a bilingual doctor reduced odd of difficulty while disability, low education, low income or recent immigration increased odds of difficulty.
McDowell et al [56]	2006	Country not applicable All settings	Systematic review and meta-analysis *n* = 24	Studies identifying at least 2 ethnic groups with one or more ADRs.	a) To review the evidence for ethnic differences in susceptibility to adverse drug reactions (ADRs) to cardiovascular drugs	• The relative risk of angio-oedema from angiotensin converting enzyme (ACE) inhibitors in African Americans compared with non-African American patients was 3.0 (95% CI 2.5 to 3.7) • The relative risk of cough from ACE inhibitors was 2.7 (1.6 to 4.5) in East Asian compared with white patients • The relative risk of intracranial haemorrhage with thrombolytic therapy was 1.5 (1.2 to 1.9) in African Americans compared with non-African American patients
Metersky et al [57]	2011	US Hospital Setting	Retrospective chart review *n* = 102,623	Ethnic minority. African Americans and Caucasians	a) Determine racial disparity in the frequency of adverse events in the Medicare Patient Safety Monitoring System	• The risk-adjusted odds of African American patient suffering a hospital-acquired infection, or an adverse drug event compared with a Caucasian patient were 1.34 (95% CI, 1.17–1.55) and 1.29 (95% CI, 1.19–1.40), respectively. • The risk-adjusted odd ratios were 0.94 (95% CI, 0.85–1.04) and 0.94 (95% CI, 0.78–1.13) for general events and procedure-related adverse events, respectively.
Nwachukwu et al [58]	2010	Country not applicable Hospital setting	Systematic Review *n* = 9	Ethnic minorities	a) To assess if the minorities have more complications than whites do after total hip re-placement and total knee replacement.	• Racial and ethnic minority groups appear to have a higher risk for early complications (those occurring within ninety days), particularly joint infection, after total knee replacement. • There was no significant difference between African Americans and Caucasians for infection rate associated with total hip replacement.
Okoroh et al [59]	2017	US Setting not applicable	Systematic review *n* = 24	Ethnic minorities	a) Explore differences in reporting race/ethnicity in studies on disparities in patient safety assess adjustment for socioeconomic status, comorbidity, and disease severity	• Eight studies included race/ethnicity in baseline characteristics and adjusted for confounders. • Hospital-level variations were infrequently analysed. • The evidence on the existence of disparities in the adverse events was mixed.
Patel et al [60]	2002	Canada Setting not specified	Mixed method study – three group of participants. Grp 1: 8 Kenyan and 5 Canadians. Grp 2: 25 English, 7 East Indians and 16 Greek parents. Grp 3: 8 English, 7 East Indians and 16 Greek	Different ethnic and cultural backgrounds.	a) Investigate and characterize the errors in cognitive processes deployed in the comprehension of procedural texts found on pharmaceutical labels by subjects of different cultural and educational backgrounds.	• All participants read and interpreted the preparation instructions for the ORT correctly, regardless of cultural background and level of formal education. • Only three of the eight (37.5%) Kenyan mothers were able to correctly administer the treatment. • The Canadian mothers were more accurate in their administration of the treatment. • Overall, cultural and educational background appeared to be only weakly related to the accuracy of dosage and administration • All groups of participants had considerable difficulty in interpreting the instructions
Raynor [61]	2016	US Clinical setting: Paediatric	Survey (*n* = 36)	Spanish and Arabic speaking	a) Identify factors affecting care in NES patients and families	• Fifty percent respondents answered that hat they did not know why they were seeing that provider, did not understand the tests, or had difficulty with interpreters. • Barriers to communication can lead to adverse medical outcomes, poor compliance with therapy, and poor understanding of medical conditions.
Romano et al [62]	2003	US Hospital setting	Quantitative analysis of 1995–2000 Healthcare Cost and Utilization Project (HCUP) Nationwide Inpatient Sample (NIS). Sample size not available	Population wide.	a) To presents national data on the incidence of Patient Safety Incidents over time and their association with patient and hospital characteristics.	• African American inpatients had a higher risk of most medical and nursing-related postoperative complications (such as decubitus ulcer; infection following infusion, injection, or transfusion; and postoperative physiologic and metabolic derangements, thromboembolism, and sepsis). • Mortality-related events and the rarest sentinel-event indicators were similarly frequent across racial/ethnic categories
Samuels-Kalow et al. [63]	2013	US Hospital setting – Pediatric ED	Prospective observational study (*n* = 146)	Spanish speaking.	a) To examine the relationship between language and discharge comprehension regarding medication dosing.	• Fifty-four percent of Spanish-speaking parents had a dosing error, as compared with 25% of English-speaking parents (odds ratio [OR], 3.7; 95% confidence interval [CI], 1.6Y8.1). • Half of the Spanish-speaking parents discharged in English (discordant discharge) had a dosing error, as compared with 62% of those discharged in Spanish. • Spanish-speaking parents were significantly more likely to have a dosing error (odds ratio, 3.7; 95% confidence interval, 1.6Y8.1), even after adjustment for language of discharge, income, and parental health literacy (adjusted odds ratio, 6.7; 95% confidence interval, 1.4Y31.7)
Schwappach et al [64]	2012	Switzerland Community setting: Pharmacy	Cross-sectional survey (*n* = 498)	Foreign language patients	a) Investigate Swiss public pharmacists’ experiences and current practices in counselling foreign-language patients (FL) with a focus on patient safety and to identify needs for subsequent improvement activities. b) Examine whether frequent experience of communication barriers is associated with pharmacists’ satisfaction with quality of care provision.	• Approximately 10% of pharmacies reported that they at least weekly fail to explain the essentials of drug therapy to FL patients • Ad-hoc interpreting by minors is also common for a considerable number of pharmacies (26.5% reported at least one weekly occurrence) • Tools for supporting communication with FL patients are used only infrequently and by a minority of pharmacies – with printing information in foreign language as the most common tool. • The main strategy used by pharmacists to improve the quality of medication counselling for FL patients was the systematic employment of multilingual staff.
Shadmi et al [65]	2013	Israel Hospital setting	Prospective Observational study (*n* = 385)	Russian speaking patients	a) Examine the quality of care transitions of minority patients (immigrants) versus the general population and assess the association between in-hospital provider–patient communication and the quality of minority care transitions.	• Russian speakers reported lower scores for understanding the purpose of taking their medications.
Shen et al [66]	2016	US Hospital setting	Cross-sectional study of hospital discharges related with Patient safety indicators (n = 3,052,268)	Ethnic minority	a) Are minority patients weremore likely to incur adverse PSIs than their White counterparts; b) Are patients with Medicaidor uninsured were more likely to incur adverse PSIs than patients covered by private insurance.	• As compared with White patients, African American patients were more likely to experience pressure ulcer, postoperative hemorrhage or hematoma, and post-operative pulmonary embolism (PE) or deep vein thrombosis (DVE). • Asian/Pacific Islander patients were more likely to experience pressure ulcer, post-operative PE or DVT, and two obstetric care PSIs. • Hispanic/Latino patients were more likely to experience post-operative physio-metabolic derangement and accidental puncture/ laceration.
Stockwell et al [67]	2019	US Hospital Setting	Multisite investigation using record review (*N* = 3790)	Ethnic minorities identified in records	a) To understand patient safety disparities not been previously identified in the pediatric inpatient environment by measuring rates of clinically confirmed AEs	• Compared with hospitalized Caucasians children, hospitalized Latino children experienced higher rates of all AEs (Latino: 30.1 AEs per 1000 patient days versus white: 16.9 AEs per 1000 patient days; *P* ≤ .001), preventable AEs (Latino: 15.9 AEs per 1000 patient days versus white: 8.9 AEs per 1000 patient days; *P* = .002), and high-severity AEs (Latino: 12.6 AEs per 1000 patient days versus white: 7.7 AEs per 1000 patient days; *P* = .02). • No significance difference was observed in other groups.
Suurmond et al [17]	2010	The Netherlands Hospital setting	Qualitative semi-structured interviews (*n* = 12) for a total of 30 cases	Foreign language speaking	a) Explore the characteristics of in-hospital care and treatment of immigrant patients to better understand the processes underlying ethnic disparities in patient safety.	• Health care professionals preferred ad hoc interpreters or no interpreter at all. • There was limited availability of translated documents for patients who do not speak or read Dutch. • Lack of insurance was identified as a barrier to receiving appropriate care. • Organisations shortcomings to understand genetic and physical characteristics of migrants increases the risk of patient safety events. • Presumptions about the cultural background of the patient resulted in an adverse event or patient safety event.
Thomas et al [68]	2010	Australia Hospital setting	Retrospective analysis of 4751 charts	Culturally and linguistically diverse *Those born outside Australia /New Zealand, non-English speaking, non-Caucasian and refugees	a) Determine whether CALD parameters, including country of birth, race, primary language spoken, need for an interpreter and refugee status are independent predictors of obstetric or neonatal outcomes.	• Use of interpreter services was associated with a reduced likelihood of an adverse outcome (*P* = 0.015),. • No significant difference observed between adverse outcomes and refugee status.
Timmins et al [69]	2002	US All settings	Systematic review (n = 14)	Latinos * all persons living in the United States whose origins can be traced to the Spanish- speaking regions of Latin America, including the Caribbean, Mexico, Central America, and South America”	a) Provide knowledge for providers and institutions in devising effective strategies for bridging the language barrier.	• Health care providers need to educate themselves and their institutions about the laws and regulations that address language access in their own particular setting • Bilingual fieldworkers need to be trained appropriately to be used for bridging the communication gap.
van Rosse et al [70]	2016	The Netherlands Hospital setting	Mixed method study Record review (*n* = 567) Patient questionnaire (*n* = 576) Qualitative data (*n* = 17)	Ethnic minority background	a) At which moments during hospitalization do language barriers constitute a risk for patient safety? b) How are language barriers detected and reported in hospital care? c) How are language barriers bridged in hospital care? What is the policy and what happens in practice?	• In 30% of the patients that reported a low Dutch proficiency, no language barrier was documented in the patient record. • Relatives of patients often functioned as interpreter for them and professional interpreters were hardly used. • Drop-out of protocolised name and DOB check was observed among people with low Dutch proficiency. • Language barrier threatened patient safety during daily nursing tasks (i.e. medication administration, pain management, fluid balance management) and patient–physician interaction concerning diagnosis, risk communication and acute situations.
van Rosse et al [71]	2014	The Netherlands Hospital setting	Prospective cohort study (*n* = 763 Dutch patients and 576 ethnic minority patients)	Ethnic minority background	a) To compare incidence, type, nature, impact and preventability of AEs during hospitalisation of Dutch patients with those of ethnic minority patients. b) To assess the extent to which patient-related determinants (language proficiency, health literacy, education and religion) are related to the incidence of AEs among Dutch and ethnic minority patients.	• No significant difference in the incidence of AEs: 11% (95% CI 9 to 14%) in Dutch patients and 10% (95% CI 7 to 12%) in ethnic minority patients. • There was no significant difference in the incidence of preventable AEs: 3% (95% CI 1 to 4%) in Dutch patients and 1% (95% CI 0 to 2%) in ethnic minority patients • Low language proficiency, inadequate health literacy and low educational level did not increase the risk of an AE.
van Rosse et al [70, 72]	2016	The Netherlands Hospital setting	Mixed method study Document analysis (*n* = 20 cases) Observations (n = 3 cases) Qualitative interviews (n = 12)	Ethnic minority background	a) Explore the potential roles that relatives take on themselves and their influence on patient safety of hospitalised ethnic minority patients.	• Apart from fulfilling their usual role as a visiting family member, relatives often took on the role of the interpreter, the patient and the care provider. • Four roles can help optimise quality and decrease safety risks for the hospitalised patient but can also increase patient safety risks. • Good understanding between the healthcare provider(s) and the relatives tended to increase patient safety.
Wasserman et al [73]	2014	US Hospital setting	Mixed method study Analysis of 39,133 AEs from char review Interpreter analysis of 28 incidents Qualitative interviews (sample not provided)	Low English proficiency (LEP)	a) To describe the development, content, and testing of two new evidence-based Agency for Healthcare Research and Quality (AHRQ) tools for LEP patient safety.	• Integration of interpreters is a complex process requiring staff training and organizational change. • LEP patients have better safety outcomes when interpreters are used but the uptake of the interpreters by health professionals is not optimal. • Inconsistent collection of patient data on race, ethnicity, and language greatly affects understanding the role of language and culture in patient safety events. • Majority (60%) of the interpreter pilot related events were related to misuse of interpreter services.
White et al [12]	2012	Australia Primary healthcare setting	Focus groups (*n* = 2) with Six Chinese & 11 Vietnamese	Chinese and Vietnamese immigrant	a) How aging home medicine review (HMR)-eligible Chinese and Vietnamese Australians who have never received a HMR manage their medicines; b) To what extent they are aware of the existence of this service c) How likely they might be to accept and receive a HMR in the future.	• Chinese participants had doubts about the effectiveness of prescribed medications, fear of generic brands, fear of taking too many medications and a lack of medicine information contributing to non-adherence and confusion. • Vietnamese patients, although having some concerns with medications did not show non-adherence to dosing regimen and voice strong respect for GP. • Both groups reported difficulties locating a pharmacist who spoke their native language, which contributed to an increased unmet need for medicine information.

### Study quality

Overall, the included studies varied against the quality assessment criteria. Most studies achieved the total possible score for criteria relating to: statement of aims, objectives or goals; study design being appropriate to address research aims; format of data collection tool to address research aims; method of data analysis; and discussing strengths and limitations. Most studies achieved a nil score against the quality criteria for the involvement of consumers or stakeholders in the research design and conduct. Only two studies described any involvement of consumers or stakeholders in the process of study design and conduct [46, 51]. Our findings highlights lack of reporting of engagement of stakeholders and ethnic minority consumers in research that concerns ethnic minority groups. The included studies provided evidence around the following key areas: I) link between patient safety events and the description of ethnic minorities in the given context; II) the disparity in iatrogenic infections, complications and medicine-related safety events relating to the process of health care; and III) various factors influencing safety events such as communication barriers, cultural factors.

### Review Q 1: how ethnic minority populations are conceptualised across the international literature and what are the implications of this conceptualisation in shaping our findings?

Three studies defined or included a definition of ethnic minority [26, 33, 69]. Minority populations were generally conceptualised in the following ways (1): by race, such as African Americans, Caucasians, Asians (2); by language, such as Spanish speaking, Low English Proficiency (LEP); and/or (3) country of origin. Most studies used one or two of the methods to define their ethnic minority populations of study [41, 66, 68]. Large administrative dataset studies largely used race as the key determinant [62, 66]. The methods used reflect the data available in major databases or routinely collected.

### Review Q 2: what is the evidence for patient safety events involving ethnic minority healthcare consumers internationally?

The nature and severity of adverse events and disparity in the occurrence of these events was examined in thirteen studies, primary emerging from the US. Differences between ethnic minority patients and others in safety events were predominantly examined using large administrative datasets, retrospective record reviews and systematic reviews focused on rates of (i) hospital acquired infections (HAIs), (ii) complications in care and (iii) adverse drug events (ADEs) [15, 35, 37, 39, 48, 56–59, 62, 66, 69, 71].

Five retrospective studies using large administrative datasets, three using the Patient Safety Indicators (PSIs) as outcome measures [39, 62, 66] and two using Medication Patient Safety Monitoring System (MPSMS) [15, 57] explored the rate of various HAIs and ADEs among patients from different ethnic backgrounds. These studies reported mixed results highlighting that some ethnic groups were at greater risk of some patient safety events. For example, in a review of administrative data from 16 states in the US, African Americans were reported to have 1.25 to over 1.5 times the rate of infections, postoperative sepsis, decubitus ulcers, postoperative respiratory failure, postoperative pulmonary embolism or deep vein thrombosis (PE/DVT) as Caucasians even when controlling for income level [39]. Findings also revealed that Hispanics had 1.25 to 1.50 times the rate of sepsis and physiologic and metabolic derangements as Caucasians; and Asian/ Pacific Islanders had 1.25 to 1.50 times the rate of sepsis, haemorrhage or hematoma, respiratory failure, and physiologic and metabolic derangement [39]. Further US studies reported disparities in a range of safety indicators for African American and Asian /Pacific Islander peoples [57, 66]. Yet in the Netherlands, a study of 763 Dutch patients and 576 ethnic minority patients revealed no difference in the rate of adverse events between the groups [71]. When compared to the US studies, the authors explained this as being due to the availability of equal access to care through a mandatory insurance scheme for patients [71].

The evidence relating to ADEs among ethnic minorities was mixed and was observed to depend on contextual features such as geographical, genetic and cultural features [35, 57, 59]. Metersky et al. in their retrospective review of 102,623 charts in US hospital settings found that the risk-adjusted odds of African American patients suffering an ADE compared with Caucasian patients was 1.29 (95% CI, 1.19–1.40) [57]. Baehr et al. in their systematic review observed that Asians were more prone to ADEs related to anticoagulants such as bleeding and African American to ADEs relating to diabetes agents such as hypoglycaemia [35]. The term Asian represented combined Asian/Pacific Islander group but authors did not specify any specific ethnic minority group from Asia. Baehr et al. and Okoroh et al. in their systematic reviews also noted increased risk of certain ADEs among certain ethnicities for some drugs and contributed these to genetic predisposition and overuse [35, 59].

Five systematic reviews concerning adverse events in surgical patients and ADEs were also included [35, 37, 56, 58, 59]. In their systematic review and meta-analyses of 26 studies relating to complications and mortality in surgical care, Bloo et al. found that ethnic minorities have a higher risk of complications in perioperative care as compared to the ethnic majority patients resulting in a higher incidence of pain and re-operation [37]. Some included studies in their review discussed the causative factors of the differences in the outcome and ranged from sociocultural, biological and presurgical risk factors but noted that these were mostly speculative highlighting the need for further analyses of the factors at patient, physician and system level [37].

Medicine-related problems were further discussed in eleven included studies [12, 26, 33, 34, 38, 44, 47, 51, 55, 61, 63]. with a range of multifactorial issues identified [12, 33, 47]. Dosing errors were highlighted as the most common error, especially in the context of children receiving the wrong dose by parents from ethnic minorities [47, 51, 55, 63]. Harris et al. in their cross sectional analysis of data from a randomised controlled experiment, identified that greater than 80% of the Hispanic parents made at least one dosing error related to liquid mediation dosage [47]. They observed that the rate of dosing errors was almost double for parents with LEP and low health literacy [47]. In their systematic review based in the UK, Alhomoud et al. demonstrated that most common medicine-related problems among ethnic minority group in the UK were not taking medicines as advised, non-adherence and limited knowledge of illness, its consequences and therapies [26].

### Review Q 3: what individual, service and system factors contribute to safety among ethnic minority healthcare consumers?

Several factors contributed to the risk of patient safety events in health care for ethnic minority patients, although their independent and collective contribution was not clear from work to date. Our narrative thematic synthesis identified following factors: 1) language proficiency; 2) errors associated with formal and informal interpretation; 3) misinterpretation of instructions; 4) income and insurance; 5) beliefs about illness and treatment; 6) family and friends for safety vigilance; and 7) patient-professional interactions. We found that often these factors were integrated and not readily distinguished as uniquely individual or service or system level factors. For example, issues around language proficiency were an individual issue in terms of one’s own language proficiency, but it was also about readiness or feasibility of accessing a health system without strong proficiency in a national or native language.

- Language proficiency

In their prospective incident record review study, Divi et al. identified some degree of detectable physical harm occurred in almost 50% of the LEP identified patients as compared to 30% harm among English speaking patients [41]. They defined LEP status as inability or limited ability to read, write or understand English that impacts on their ability to interact with healthcare professionals [41]. Although communication failure was detected as a factor for more than half of the adverse events occurring in LEP patients, the authors also identified the giving of questionable advice, and incorrect/inadequate interpretation and assessment of the patient by the health professional as possible causes [41]. In a paediatric setting, Cohen et al. found that Spanish speaking families who needed language assistance had a significantly higher risk of experiencing a serious adverse event [40]. But authors also acknowledged that lower socioeconomic status, lower income level and lack of insurance status may impact on their findings [40]. Identifying patients with limited language proficiency is essential to provide relevant interpreting services; however, three studies identified that often there is either an overestimation or incorrect recording of the language data [45, 70, 73]. In one retrospective record review of adverse or sentinel events in the US, the authors identified that medical records data relating to language was only entered correctly 30% of the time [73]. In another record review study conducted in the Netherlands, 30% of patients identified as low Dutch proficient, had no mention of a language barrier or language proficiency documented in their medical record [70].

b) Errors associated with formal and informal interpretation

The review revealed that use of professional interpreting services was free in most developed countries. However, their use by healthcare professionals was limited with many choosing to use ad hoc interpreters, such as carers, family members or people other than the qualified interpreters, or no interpreters [17, 54, 68, 70]. In their qualitative study exploring the role of relatives of ethnic minority patients for patient safety in hospital care, van Rosse et al. noted that use of relatives as interpreters can increase the patient safety risk by inadequately interpreting information to the patient but can also decrease the patient safety risk when the use of interpreter is not feasible [72]. Relying on patient/carer interpretation during the care process is sometimes required and this can reduce reliance on professional services, but is problematic due to the code of confidentiality thus limiting communication [46]. The key risks resulting were poor communication, inadequate patient assessment, poor patient understanding of prescribed treatment and inadequate warning about clinical risks associated with treatment [46, 72, 73]. Two studies examining use of various types of interpreters and their impact on errors of potential clinical consequences were also included [42, 43]. Flores et al. analysed 13 interview transcripts consisting of six trained medical interpreters and seven ad hoc interpreters [42]. They found 396 interpreting errors with an average of 31 errors per encounter. The most common error types were omission, false fluency, substitution, editorialisation and addition [42]. They found that errors with potential clinical consequence to be significantly higher when ad hoc interpreters were used compared to the use of trained medical interpreters (77% vs 53%) [42]. However, this study did not discuss if adverse events occurred. In another cross-sectional study involving two paediatric hospitals, audio taped interpreter encounters (*n* = 57) revealed that the use of ad hoc or no interpreters was related to double the errors of potential clinical consequence when compared to using trained medical interpreters [43]. They noted that recency of practice was linked with less errors than the years of experience for the trained interpreters [43].

c) Misinterpretation of instructions

Patient safety events resulting from the misinterpretation of medication prescription labels and misunderstanding of discharge instructions were more commonly reported among ethnic minority patients in nine studies [17, 36, 47, 50, 51, 55, 60, 63, 65]. In their mixed-method study involving 308 patients (203 with LEP), Karliner et al. used principle discharge diagnosis, medication outcomes (category, purpose, combined category and purpose) and follow up appointment type as markers for understanding discharge instructions [50]. They observed that patients with LEP had lower odds of understanding medication category (0.63) and combined medication purpose and category after adjusted analysis reflecting some difficulty in understanding discharge instructions [50]. Masland et al. in their analysis of California’s 2007 Health Interview Survey (*n* = 48,968) found that LEP increased the odds of difficulty in understanding the prescription instructions three times for Central Americans, Mexicans and Koreans and four times for Chinese. They also found that low income (OR1.7), low education (OR1.6) and recent migration (OR1.5) increased the chances of difficulty in understanding prescription [55]. While having a bilingual doctor reduces the odds of difficulty in understanding of prescriptions for Mexicans, Koreans and Vietnamese, it does not for Chinese and Central Americans [55]. The authors explain that high level of linguistic diversity within the Central Americans and Chinese may have impact on truly finding a language concordant bilingual doctor [55]. Two studies also identified that health professionals’ self-identified capacity to provide instructions in patients language can also lead to increased risk of safety events though no quantitative data was available [53, 73].

d) Income and Insurance

Six included studies discussed impact of income or insurance on patient safety with five studies emerging from the US [37, 39, 57, 66, 69, 71]. Coffey et al. and Shen et al. in their review using large administrative dataset and PSI as outcome measures noted that the rate of PSIs for low income group and the patients with Medicaid was higher than for high income group or those with private health insurance [39, 66]. These studies compared the impact of insurance or income independently. Mix results were obtained when they were studied together with ethnic status demonstrating increased risk for African Americans. Shen et al. observed that there was no racial disparities for pressure ulcer among patients with Medicaid, but African Americans with private insurance were more likely than Caucasians to incur pressure ulcer [66]. Coffey et al. studied the impact of income and racial group and noted that African Americans had higher rate for PSIs than Caucasians in all community income groups [39]. Authors noted a range of factors that may contribute to this relationship such as hospital resources [39, 66]. In settings with universal health insurance or access such as The Netherlands, there appeared to be fewer differences in safety events between ethnic minority and other patients but a lack of studies examined the role of income or private health care coverage on safety events outside of US settings [71].

e) Beliefs about illness and treatment

Suurmond et al. and Masland et al. noted that consumers from ethnic minorities have different beliefs about illness and treatment to that of the health professionals depending on their cultural and religious beliefs [17, 55]. Six other studies discussed the differences in the beliefs about illness and treatment and its impact on safety events [12, 26, 33, 38, 52, 55]. These were discussed in the context of medication adherence, compliance to treatment recommendations, but no quantitative data was available to measure the impact on patient safety events. The impacts observed varied and depended on population and setting. A systematic review examining medicine-related problems among ethnic minority population in the UK identified that beliefs about illness and treatment based on religious practices may cause safety events such as not taking medication as prescribed [26]. A qualitative study expanding on this identified that religious practices such as patients fasting for religious purposes alter their medications by changing the time or completely stopping it without consulting their doctor [33]. In their mixed methods before-after study using focus groups and surveys, Cantarero-Arevalo et al. observed that ethnic minority consumers often feel isolated in a new country due to language and cultural differences resulting in mistrust with health professionals and non-compliance with medicine and treatment [38]. Two studies discussed interventions, using bilingual workers from same background to that of the consumers providing culturally appropriate education program to ethnic minorities, to improve medication adherence and improve knowledge of treatment [38, 52]. A pre- and post- intervention survey of the Korean adults (*n* = 68) receiving education about medication prescriptions by Korean speaking pharmacy students demonstrated the adults significantly increased their understanding of medication directions and had improved trust with pharmacists [52]. Another mixed method study evaluated culturally competent education provided by bilingual fieldworker from the same culture and concluded improved knowledge of medicine-related problems [38]. However, the sample size was small (*n* = 8) to demonstrate generalisability. Neither of these interventions demonstrated quantitative impact on patient safety events.

f) Family and friends for safety vigilance

Families and friends were identified in having an important role for safety vigilance of their loved ones in two studies. Van Rosse et al. observed that patients from ethnic minorities were more often accompanied by families or friends compared to Dutch born patients during hospitalisation and voiced concerns to the health professionals which enhanced their safety [71]. Another qualitative study of 80 semi structured interviews with consumers from South Asian or Middle Eastern backgrounds in the UK reported that participant often stopped their medications on advice of family or friends, consumed non-prescription medication on advice of family or friends, and also shared their medicines with family members resulting in double dosing for some [33]. van Ross et al. in their study conclude that patient safety can be enhanced by optimising collaboration with the family and friends who are willing to take part in the care process [71]. Goenka, in her literature review, provided one such example of collaboration – Family centred round (FCR) - in a hospital setting to improve collaboration with families of LEP patients [45]. However, they did not evaluate the effectiveness of the program on patient safety.

g) Patient-professional interactions

Ethnic minority patients’ interactions with health professionals and systems were discussed in seven studies with focus on bias and lack of communication resulting in inappropriate care with potential for a patient safety event [12, 17, 26, 33, 36, 63, 64]. Health professional’s presumption about the desire of ethnic minority consumers to engage in their care or their level of understanding may result in unsafe care. In their prospective observational study of 210 paediatric emergency department discharges, Samuels-Kalow et al. observed that when health professionals were providing information regarding pain killers (acetaminophen) to patients, they gave different information to English speaking and Spanish speaking parents [63]. In their systematic review of medicine-related problems in ethnic minority patients in the UK, Alhomoud et al. noted an underestimation of patients desire for information and engagement in decision making by health professionals [26]. In a mixed method study using interviews with consumers from four different ethnicities (*n* = 18), authors noted that the lack of communication between various departments within a hospital as well as with community practitioners such as general practitioners can impact on care such as ceasing a patient’s medicines prescribed by a GP during hospitalisation [36]. This frequent change applies to all populations but potentially this is a greater threat to patients from ethnic minorities due to various other compounding factors, such as greater investment of time required to build relationships and engagement with ethnic minority consumers [25].

## Discussion

Our review findings provide substantial evidence to suggest that people from ethnic minorities are vulnerable to a higher rate of patient safety events in the hospital and community setting compared to the mainstream population. Specifically, included studies showed that ethnic minorities experienced higher incidences of healthcare associated infections, dosing errors, ADEs and complications resulting from their care [35, 37, 39, 57, 66]. Ethnic minority populations being more likely to experience unsafe health care may compound or contribute to already existing inequities in health outcomes. Importantly, this review also highlights the limited socio-cultural data available in health systems internationally and the implications of this for establishing healthcare inequalities.

**Fig. 2:**
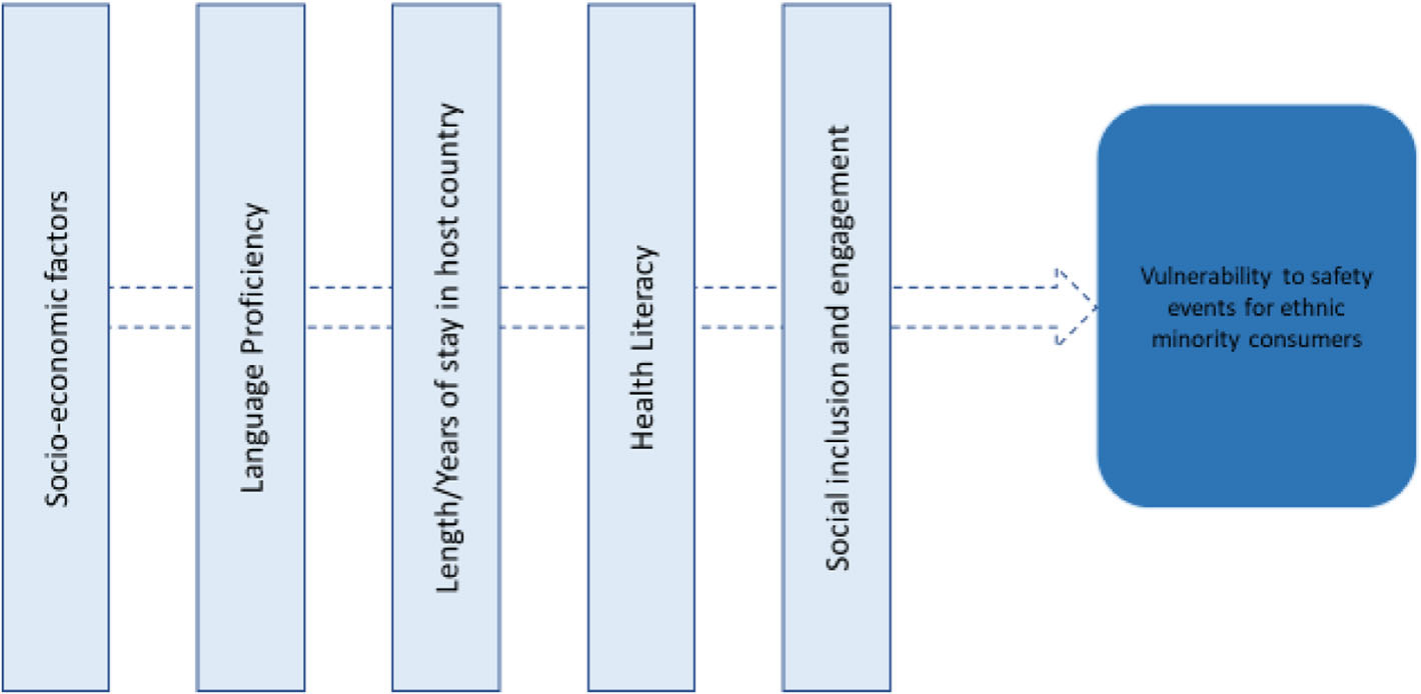
Conceptual model for vulnerability to safety events for ethnic minority consumers

Factors contributing to the increased threat to ethnic minority populations include socioeconomic factors such as income, insurance and education; language proficiency; health literacy; length of stay in the host country; feeling of alienation and distrust for the health professional, services and systems; and, engagement [38, 39, 41, 55, 64, 66]. Included studies mostly studied these factors independently of each other with little discussion of any relationship between them. In the limited evidence available of this relationship, it was apparent that LEP and health literacy had implications for understanding prescriptions for medication [55]. Health literacy has been explored extensively in relation to poor health outcomes for ethnic minority patients, but there is limited evidence regarding its role in, and interactions with, factors linked to patient safety events [74]. A conceptual model (Fig. 2) has emerged from our review that identifies the major factors that heighten the vulnerability of ethnic minority patients to patient safety events. Further knowledge regarding the cumulative impact of these factors on patient safety events is required as well as better understanding of their relationship with each other and the level of impact each factor may have on potential patient safety events.

Health systems change towards providing culturally competent care by hiring more interpreters or using bilingual health professionals were commonly cited in the reviewed articles [51, 55, 64]. Yet studies to date have mainly explored organisational and staff cultural competence in the context of improving access to care and communication rather than preventing unsafe care specifically [75, 76]. The role of bilingual healthcare staff in positively addressing language and culture was identified in a number of studies [51, 58, 64]. Bilingual staff may improve communication and are highly valued by organisations and patients alike as they speak the patients’ language and understand their culture context [25, 77]. The evidence from the present review suggests that use of interpretation through a range of means is valuable in addressing some patient safety issues but may also leads to others.

Mobile technologies that can translate for interpretation are recognised as a potential solution to overcome language barriers; research indicates ethnic minority consumers use this technology [25]. Google Translate, a web-based tool, is available but low translation accuracy of medical terminologies has the potential for distress and harm [78]. Current applications such as CALD- Assist (an Australian application) that uses pre-recorded text words and phrases along with pictures and videos for allied health and nursing staff to facilitate communication when interpreters are not available or not practical [79] notwithstanding the applications have shown improved satisfaction for communication between consumers and health professionals. More research is needed to understand the patient safety implications of them.

The importance of the active participation and engagement of healthcare consumers both in care and study design is recognised through this review as contributing to safety outcomes and research quality. The relationship between consumer involvement in healthcare and enhanced health outcomes including patient safety is well-established and reinforced through this review [80]. The Australian Commission of Safety and Quality in Health Care (ACSQHC) identifies consumer engagement strategies as central to improved health and healthcare safety outcomes for consumers [21, 81]. Recent studies has also highlighted the role of patient and carer engagement among ethnic minority populations as critical for enhanced healthcare [25, 77]. Consumers can act as safety buffer by identifying when the care is not adequate or unsafe [82]. Included studies in the present review reflect this demonstrating that family members and carers can contribute to safety by voicing their concerns to health professionals and advocating for the patient [71].

### Implications

Policy organisations focused to improve health care quality internationally identify ethnic minorities as a priority group but specific guidance about reducing patient safety events is limited [83, 84]. Key implications from this review relate to the vital role of family and carers in supporting safe care. Despite recognition of the need for enhanced consumer engagement among ethnic minority consumers, few service and system level support structures exist to support engagement with patients or their family members [14, 26]. Strategies utilised by the wider patient population, such as checklists or consumer toolkits designed to assist in asking health professionals questions and raising concerns during their care, are under-utilised in ethnic minority populations [81]. Engagement strategies that take into account the nuance differences between ethnic minority consumers are lacking and involving ethnic minority consumers can help develop relevant engagement strategies [25]. Co-design approaches to adapt and develop suitable engagement strategies may be of value to address this gap. A key consideration is how to ensure that approaches to enhance family and carer engagement are explored.

We also found deficiencies in routinely collected healthcare data. Our synthesis of the included studies showed insufficient data regarding factors, that collectively create an individual’s ethnic profile, with most data sets relying on information about country of birth, language spoken and in some instances race to classify ethnic minority individuals. Classifications such as ‘Asian’ or ‘Caucasian’ can be deemed to be homogenous and fails to capture the range of different ethnic minorities included under either of the terms [85]. Given the evidence of multiple patient safety threats for ethnic minority groups, establishing an individual’s ethnic profile in greater detail is essential so as to fully understand if particular patient safety issues are in play and to develop focused interventions.

Several authors commented on the superficial identification of ethnic minority patients and recommended systems be strengthened to allow complete identification of ethnic minorities [45, 70]. There is a need to register more specific socio-cultural data. It is recognised that nuanced differences exist between various ethnic minority groups [86] and collection of superficial indicators to identify ethnic minority population may not provide a full picture of cultural and social factors shaping practices and understanding of health of a particular ethnic minority group. The Australian Bureau of Statistics (ABS) recommends that diverse variables be used to identify consumers from different ethnic minorities [87]. Health services often only collect data relating to language, country and interpreter use [87, 88]. In the US context, Wilson-Stronks et al. recommended level of health literacy, cultural needs, dietary needs and socioeconomic variables be collected along with language and religion [85]. Similarly, Lopez et al. recommended an automated standard system -designed in consultation with ethnic minority consumers - for collecting data concerning ethnic minority and language proficiency [89]. They saw this as a way to reduce the disparity in quality and safety of health care [89].

### Limitations

This review has some limitations. The diversity of the definitions used to determine the ethnic minority population shaped the findings. For example, in United States, ethnic minorities were primarily defined based on the race or the first language, with many studies focused to differentials between African Americans, Hispanic and Caucasian populations [57, 66]. This differentiation resulted in most studies from the US using large data to examine the impact of insurance or income to the disparity in safety events [39, 66]. This also led to focus on language, especially in context of Hispanic population, as a factor for the disparity [40]. In addition, use of terms such as ‘Asian’ appeared to refer to two different population groups; in US and Australia this primarily referred to people from China or far eastern Asian countries, but in the UK this was often used in the context of people from India/Pakistan [90]. We limited our search to Organisation for Economic Co-operation and Development (OECD) countries as the ethnic minority groups are considered more comparable and thus our findings are not generalisable beyond the OECD countries. The review sought only published works which may have led to the omission of other relevant works that were not published. Grey literature including government reports were not included and this may have led to the omission of relevant information. The inclusion of five databases along with the reference lists of included articles provided a broad and varied range of data sources to identify relevant literature. Publication bias may also had impact on included studies with non-published negative findings omitted [91].

## Conclusion

Our findings indicate substantial disparity in safety events occurring between ethnic minority and mainstream populations. The nature of patient safety events to which ethnic minority populations are exposed to is dependent on the setting and minority population. Patient safety appears to be linked with the availability and use of interpreters, degree of engagement with family and carers, and the availability of systemic processes to overcome language and socio-economic barriers. Enhanced socio-cultural data across health systems is critical. Knowledge of the factors that contribute and compound to create safety for ethnic minority groups, and of the individual differences between various ethnic minority groups in terms of their healthcare safety is limited. Engaging ethnic minority consumers in the design of interventions to enhance safety is key.

## Supplementary information

**Additional file 1: Supplementary file 1** for search strategy.

**Additional file 2: Supplementary file 2** for quality assessment criteria.

## Abbreviation

ABS: Australian Bureau of Statistics
ACSQHC: Australian Commission of Safety and Quality in Health Care
ADE: Adverse drug events
CALD: Culturally and linguistically diverse
FCR: Family centred round
LEP: Low English proficiency
MPSMS: Medication Patient Safety Monitoring System
OECD: Organisation for Economic Co-operation and Development
PSI: Patient Safety Incident
UK: United Kingdom
US: United States
