# Supplementary material for: The safety of health care for ethnic minority patients: a systematic review
Source: Int J Equity Health. 2020 Jul 8;19:118. doi: 10.1186/s12939-020-01223-2 (PMC7346414; doi:10.1186/s12939-020-01223-2)
Supplement: Supplementary file 1 — Additional file 1: Supplementary file 1 for search strategy. [file 12939_2020_1223_MOESM1_ESM.docx]

**Supplementary File 1_ Search strategy**

| **#** | **Searches** |
| --- | --- |
| 1 | Cultural Diversity/ |
| 2 | Cultural Characteristics/ |
| 3 | Communication Barriers/ and language/ |
| 4 | Ethnic Groups/ or "Emigrants and Immigrants"/ or Minority Groups/ |
| 5 | Minority Health/ or Culturally Competent Care/ or refugees/ |
| 6 | or/1-5 |
| 7 | (asylum seeker* or migrant* or immigrant* or "culturally and linguistically diverse" or CALD or "Non English Speaking" or NESB or (Ethnic* adj2 patient*) or Indigenous population* or Language barrier*).mp. |
| 8 | 6 or 7 |
| 9 | exp Medication Errors/ |
| 10 | (Diagnostic Errors or Patient Safety or Iatrogenic Disease or Hospital Mortality).mp. |
| 11 | (Patient Readmission or Length of Stay or Root Cause Analysis).mp. or Patient Care/st or Waiting lists.mp. |
| 12 | 9 or 10 or 11 |
| 13 | (near miss* or close call* or Nosocomial or (Patient* adj2 safety) or adverse outcomes or adverse events or incident reports or Root Cause Analysis or RCA or sentinel event*).mp. |
| 14 | 12 or 13 |
| 15 | 8 and 14 |
| 16 | limit 15 to yr="2000 -Current" |
| 17 | limit 16 to english language |
| 18 | 17 not Cerebral adrenoleukodystrophy.mp |
| 19 | (developing countr* or third world or underdeveloped countr* or under developed countr*).mp. |
| 20 | exp africa/ or americas/ or exp caribbean region/ or exp central america/ or latin america/ or mexico/ or exp south america/ |
| 21 | exp europe, eastern/ or exp transcaucasia/ |
| 22 | antarctic regions/ or exp atlantic islands/ or exp indian ocean islands/ or exp pacific islands/ |
| 23 | New Guinea/ or asia/ or exp asia, central/ or asia, southeastern/ or borneo/ or cambodia/ or east timor/ or indonesia/ or laos/ or malaysia/ or mekong valley/ or myanmar/ or philippines/ or thailand/ or vietnam/ or asia, western/ or bangladesh/ or bhutan/ or india/ or middle east/ or afghanistan/ or iran/ or iraq/ or jordan/ or lebanon/ or oman/ or saudi arabia/ or syria/ or turkey/ or yemen/ or nepal/ or pakistan/ or sri lanka/ or far east/ or china/ or tibet/ or exp korea/ or mongolia/ |
| 24 | (Afghanistan or Africa or Albania or Algeria or Angola or Antigua or Argentina or Armenia or Azerbaijan or Bangladesh or Barbados or Barbuda or Belarus or Belize or Bhutan or Bolivia or Bosnia or Botswana or Bulgaria or Burkina Faso or Burundi or Cambodia or Cameroon or Central African Republic or Chad or Chile or Colombia or Comoros or Congo or Costa Rica or Croatia or Cuba or Czech* or Congo or Djibouti or Dominica or Dominican or East Timor or Ecuador or Egypt or El Salvador or Equatorial Guinea or Eritrea or Estonia or Ethiopia or Fiji or Gabon or Gambia or Ghana or Grenada or Guatemala or Guinea-Bissau or Guyana or Haiti or Honduras or Hungary or India or Indonesia or Iran or Iraq or Ivory Coast or Jamaica or Jordan or Kazakhstan or Kenya or Kiribati or Kyrgyzstan or Laos or Latvia or Lebanon or Lesotho or Liberia or Libya or Lithuania or Madagascar or Malawi or Malaysia or Maldives or Mali or Marshall Islands or Mauritania or Mauritius or Mexico or Micronesia or Moldova or Mongolia or Montenegro or Morocco or Mozambique or Myanmar or Namibia or Nepal or New Guinea or Nicaragua or Niger or Nigeria or Korea or Oman or Pakistan or Palau or Panama or Papua New Guinea or Paraguay or Benin or China or Peru or Philippines or Poland or Cape Verde or Georgia or Kosovo or Macedonia or Yemen or Romania or Russia or Rwanda or Saint Kitts or Saint Vincent or Saint Lucia or Sao Tome Principe or Saudi Arabia or Senegal or Serbia or Seychelles or Sierra Leone or Slovak* or South Africa or Solomon Islands or Somalia or Sri Lanka or Sri-Lanka or Sudan or Suriname or Swaziland or Syria or Tajikistan or Tanzania or Thailand or Togo or Tonga or Trinidad or Tobago or Tunisia or Turkey or Turkmenistan or Uganda or Ukraine or Uruguay or Uzbekistan or Vanuatu or Venezuela or Vietnam or Samoa or Zambia or Zimbabwe).af. |
| 25 | or/19-24 |
| 26 | 18 not 25 |
| 27 | limit 26 to (addresses or case reports or comment or editorial or letter) |
| 28 | 26 not 27 |
