# Supplementary material for: The safety of health care for ethnic minority patients: a systematic review
Source: Int J Equity Health. 2020 Jul 8;19:118. doi: 10.1186/s12939-020-01223-2 (PMC7346414; doi:10.1186/s12939-020-01223-2)
Supplement: Supplementary file 2 — Additional file 2: Supplementary file 2 for quality assessment criteria. [file 12939_2020_1223_MOESM2_ESM.docx]

**Supplementary File 2_Quality Assessment Criteria**

| **Criteria** | |
| --- | --- |
| **1** | Theoretical or conceptual underpinning to the research |
| **2** | Statement of research aim/s |
| **3** | Clear description of research setting and target population |
| **4** | The study design is appropriate to address the stated research aim/s |
| **5** | Appropriate sampling to address the research aim/s |
| **6** | Rationale for choice of data collection tool/s |
| **7** | The format and content of data collection tool is appropriate to address the stated research aim/s |
| **8** | Description of data collection procedure |
| **9** | Recruitment data provided |
| **10** | Justification for analytic method selected |
| **11** | The method of analysis was appropriate to answer the research aim/s |
| **12** | Evidence that the research stakeholders have been considered in research design or conduct. |
| **13** | Strengths and limitations critically discussed |
